# American Journal of Medical Sciences

**Published:** 1860-05

**Authors:** 


THE AMERICAN JOURNAL OF MEDICAL SCIENCES. Edited by ISAAC HAYS, M. D. Philadelphia: Blanchard & Lea. Price $5 per year.

There are numerous weekly Journals, recording the passing events of the day, containing valuable reports, and stored with useful communications from members of the profession in all parts of the country. We have also a number of monthlies, the communications to which should be of a higher and more elaborate character. But notwithstanding all these, there is a necessity for one or more Quarterlies, devoted chiefly to the analysis and reviews of the numerous works that are being issued from the medical press, together with a quarterly summary of the progress of Medicine, Surgery, Midwifery, Anatomy and Physiology, Physiological and Pathological Chemistry and Forensic Medicine. Among these we know of none that so much deserve the patronage and praise of the whole professiou as the *American Journal of Medical Sciences*.

